# Retinoic Acid: A Key Regulator of Lung Development

**DOI:** 10.3390/biom10010152

**Published:** 2020-01-17

**Authors:** Hugo Fernandes-Silva, Henrique Araújo-Silva, Jorge Correia-Pinto, Rute S Moura

**Affiliations:** 1 Life and Health Sciences Research Institute (ICVS), School of Medicine, University of Minho, 4710-057 Braga, Portugal; hugomiguelfsilva@gmail.com (H.F.-S.); henriqueeasilva@gmail.com (H.A.-S.); jcp@med.uminho.pt (J.C.-P.); 2 ICVS/3B’s-PT Government Associate Laboratory, 4710-057 Braga/Guimarães, Portugal; 3 PhDOC PhD Program, ICVS/3B’s, School of Medicine, University of Minho, 4710-057 Braga, Portugal; 4 Department of Pediatric Surgery, Hospital of Braga, 4710-243 Braga, Portugal

**Keywords:** vitamin A, retinol, respiratory system, lung specification, branching morphogenesis, alveologenesis

## Abstract

Retinoic acid (RA) is a key molecular player in embryogenesis and adult tissue homeostasis. In embryo development, RA plays a crucial role in the formation of different organ systems, namely, the respiratory system. During lung development, there is a spatiotemporal regulation of RA levels that assures the formation of a fully functional organ. RA signaling influences lung specification, branching morphogenesis, and alveolarization by regulating the expression of particular target genes. Moreover, cooperation with other developmental pathways is essential to shape lung organogenesis. This review focuses on the events regulated by retinoic acid during lung developmental phases and pulmonary vascular development; also, it aims to provide a snapshot of RA interplay with other well-known regulators of lung development.

## 1. General Introduction

Retinoic acid (RA), the active metabolite of retinol (Vitamin A), plays countless roles in different organs and tissues. In the adult, RA is crucial for immune, nervous and reproductive systems, as well as for vision and skin renewal. Throughout embryogenesis, RA acts as a potent morphogen that mediates cellular signaling and transcriptional regulation, thus modulating numerous aspects of embryo development [1,2,3]. RA levels must be tightly regulated, if not, RA can act as a teratogen, rather than a morphogen. In fact, deficiency or excess of RA causes severe congenital malformations that may affect somite, skeletal, spinal cord, limb, heart, pancreas, eye, diaphragm, and lung development [2].

Regarding lung development, RA regulates cellular events such as proliferation, differentiation, patterning, and maturation [4]. Evidently, this signaling pathway “does not walk alone”, and the interplay with other well-known regulators is critical for proper lung formation. This review highlights the role of retinoic acid signaling all through lung development, its interaction with other signaling pathways, and its impact on fetal and adult diseases.

## 2. Retinoic Acid Signaling Pathway Overview

In the blood, serum retinol travels in association with Retinol-binding protein 4 (RBP4) [5]. At the target tissues, retinol may enter the cells through two distinct mechanisms: (1) membrane transport through the Stimulated by retinoic acid 6 (STRA6) membrane transporter [6] or (2) membrane diffusion, dependent on gradient concentration, as a result of its hydrophobic structure [7]. Once in the cytosol, retinol molecules are sequestered by membrane systems and bind to Cellular retinol-binding protein 1 (CRBP1), which plays a role in vitamin A cytoplasmic trafficking [8].

Inside the cell, retinol can either be interconverted into retinyl esters, to serve as cellular storage, in a reaction catalyzed by Lecithin retinol acyltransferase (LRAT) [9]; or undergo the 2-steps oxidative pathway to generate RA for signaling. In this case, the conversion of retinol into retinal is performed by the Retinol dehydrogenase (RDH) family, primarily by RDH10 [10,11]. Alcohol dehydrogenase (ADH) enzymes convert retinol into retinal, only when retinol is not associated with CRBP1 [12]. This reversible reaction is mediated, in the opposite direction, by Dehydrogenase/Reductase SDR family, particularly DHSR3 [11,13]. The second oxidative step is the irreversible conversion of retinal into RA, catalyzed by Retinaldehyde dehydrogenase (RALDH). There are three main subtypes RALDH1, RALDH2, and RALDH3 [14,15] that display diverse expression patterns throughout organogenesis [16]. RA intracellular levels must be tightly controlled to maintain the appropriate RA concentration and consequent tissue distribution. Cytochrome P450 (CYP) subfamily 26 of enzymes degrade the excess of RA to avoid detrimental effects [17]. Among the three subtypes (CYP26A1, CYP26B1, and CYP26C1), CYP26A1 is particularly important during embryonic development [17].

**Figure 1:**
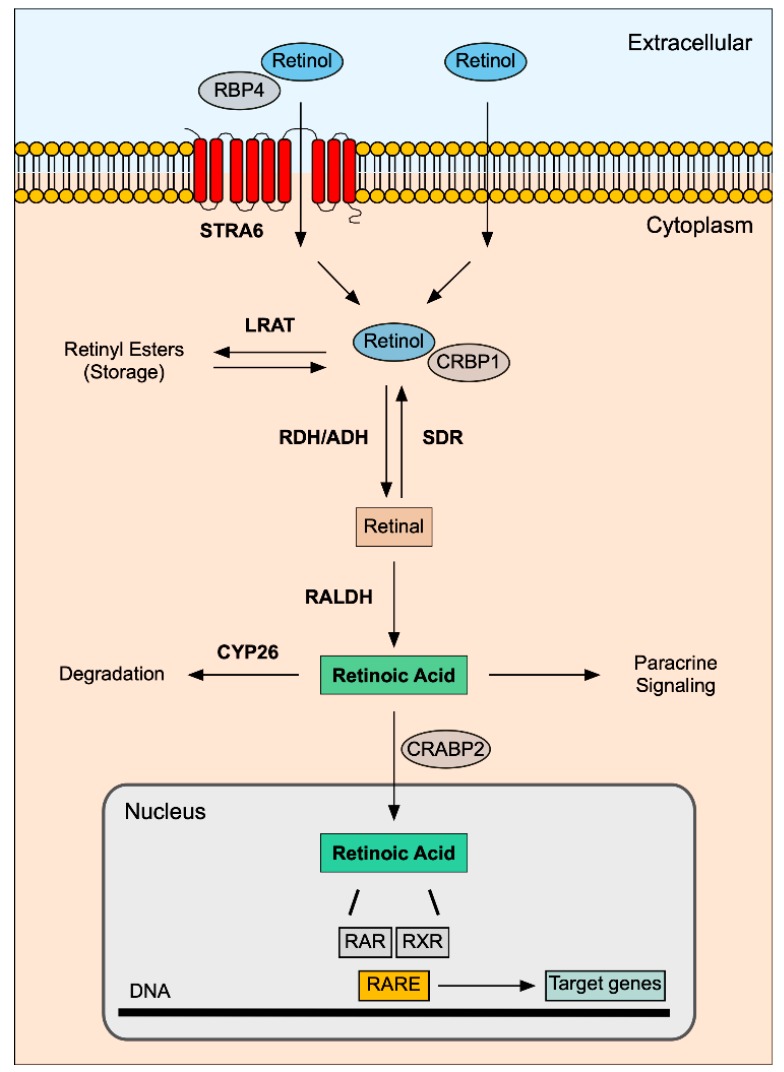
Retinoic acid signaling pathway. Retinol travels in the bloodstream in association with RBP4. Retinol can enter the target cell handled by STRA6 (binds RBP4 with high affinity), or by membrane diffusion. Inside the cell, retinol travels in association with CRBP1. Retinol can be interconverted into Retinyl esters (cellular storage) by LRAT or be transformed into retinal by RDH/ADH enzymes. Afterwards, retinal is oxidized to retinoic acid by RALDH enzymes. CYP26 degradative enzymes tightly regulate the intracellular levels of retinoic acid. Retinoic acid can travel to the neighboring cells to act in a paracrine fashion or be transported to the nucleus bound to CRABP2. In the nucleus, retinoic acid interacts with RAR and RXR nuclear receptors, which form a heterodimer that recognizes RARE sequences in the promoter region of target genes, thus modulating transcription.

Either acting on the producing cell (autocrine signaling) or the receiving cell (paracrine signaling), RA is transferred into the nucleus by Cellular retinoic acid-binding protein 2 (CRABP2) [18]. Once inside the nucleus, RA binds to specific nuclear transcription factors named Retinoic acid receptors (RARs), of which there are three main subtypes RARα, RARβ, and RARγ [19]. On its turn, RARs bind to Retinoid X receptors (RXRα, RXRβ, and RXRγ) to form heterodimers [19] that recognize specific DNA sequences (Retinoic acid response element - RARE) in the promotor region of RA target genes [20]. In the canonical model, RA-RAR-RXR complex binds to RAREs and induces conformational changes that promote the replacement of co-repressors by co-activators. Nuclear receptor co-activators (NCOA) recruit Histone acetylases and trithorax proteins, which trigger chromatin relaxation and induce gene transcription [1]. In contrast, when RA is absent, RAR-RXR heterodimers bind to RARE and recruit Nuclear receptor co-repressors (NCOR). NCOR recruits repressive factors such as Histone deacetylases and Polycomb repressive complex 2. This interaction results in chromatin condensation and gene silencing [1]. In Figure 1, the main events of the RA signaling pathway are illustrated.

## 3. Lung Development and Retinoic Acid

Lung development is a highly coordinated and multistage process governed by mechanical, anatomical, and biochemical events. In humans, it begins around the fourth week after conception and continues into the post-natal period approximately until 22 years of age [21,22,23]. The mature lung displays a tree-like tubular system connected to the trachea and a highly branched vascular system. The conducting (proximal) airways differ from the respiratory (distal) airways. In fact, towards the distal portion of the lung, the airways become progressively smaller/narrower, ending in the alveolar region where gas exchange occurs [24,25].

In humans, lung organogenesis initiates with the appearance of an endodermal bud on the ventral side of the anterior foregut around the fourth week of gestation. The endodermal evagination leads to the formation of the two lung buds and trachea, surrounded by mesoderm and a vascular network [25]. Lung epithelium is endoderm-derived and lines the airways and the alveoli. The conducting airways are generated by a highly regulated dichotomous branching morphogenesis commencing on the 5th week of gestation, giving rise to a complex network with numerous terminal branches. The continuous bifurcation of the epithelial bud tips relies on the close interaction between epithelium and mesenchyme. The mesenchyme at the tips is particularly important since it contains precursor cells that will differentiate into smooth muscle that stabilize the cleft between the newly formed buds. At the same time, diffusible signals act in both compartments, orchestrating lung organogenesis processes [26,27,28]. The alveolar cellular differentiation begins between the 16th and the 24th week of gestation. During this phase, bronchial epithelial cells differentiate into alveolar epithelial cells type 1 (AEC1) and type 2 (AEC2), also known as type 1 and type 2 pneumocytes, respectively. AEC2 act as AEC1 progenitor cells and are responsible for surfactant production, whereas AEC1 are responsible for gas exchange. From the 24th week onwards, tissue projection into the distal airspaces gives rise to structures resembling sacs, the primitive alveoli, lined with AEC1 and AEC2. Although the alveoli are formed at birth, their maturation begins approximately five weeks after birth, with the formation of a fine air-blood barrier composed by a thin epithelial sheet, an endothelial layer, and a basement membrane. Even though in the first years of life, the alveolar size remains constant, in adolescence, with the enlargement of the thoracic cage, it increases [29,30,31].

**Figure 2:**
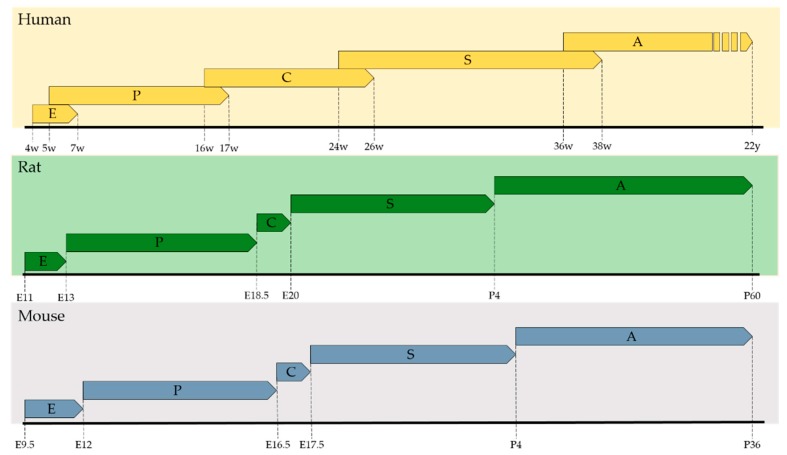
Lung developmental phases. Time scale of human, rat and mouse lung developmental phases (E = Embryonic; P = Pseudoglandular; C = Canalicular; S = Saccular; A = Alveolar). The phases are defined largely by morphological standards and may overlap in time. Adapted from [30].

The crosstalk between the epithelial and mesenchymal compartment is conveyed by several diffusible signals from multiple signaling pathways including Sonic Hedgehog (SHH) [32], Wingless-related Integration Site (WNT) [33], Transforming Growth Factor β (TGFβ), Bone Morphogenetic Protein (BMP) [34], Fibroblast Growth Factor (FGF) [35], Hippo [36], and Retinoic Acid [4], just to name a few. In the next sections, we will dissect the role of RA throughout the five lung developmental phases, namely, embryonic, pseudoglandular, canalicular, saccular, and alveolar (Figure 2), and also in lung vascular development.

### 3.1. Embryonic Phase

The respiratory system is specified from the anterior foregut endoderm approximately at embryonic day 9.5 (E9.5) in mouse and around the 4th week of gestation in humans [30]. Initially, the anterior foregut begins as a sole epithelial tube surrounded by mesoderm. Lung and trachea specification begins with the establishment of a localized expression domain of the transcription factor NKX2.1 (also known as TTF1) in the ventral wall of the anterior foregut [37]. The expression of *nkx2.1* on the ventral side, distally to the rudimentary trachea, leads to the evagination of the two primary lung buds [25]. The epithelial outgrowth of the primary buds is supported by diffusible signals from the mesoderm tightly regulated both in time, dosage, and space. Briefly, WNT2 and WNT2b expression specify NKX2.1^+^ respiratory endoderm progenitors in the ventral anterior mesoderm that surrounds the anterior foregut endoderm [38]. The expression of NKX2.1 also depends on BMP signaling activation. Endodermal SHH regulates BMP4 expression in the ventral mesenchyme surrounding the ventral foregut, mainly through the transcription factors FOXF1, GLI1, and GLI3. BMP signaling represses the expression of the transcription factor SOX2 (that promotes esophageal fate), thus enabling NKX2.1 endodermal expression [39]. After respiratory lineage specification, FGF10 mesodermal expression requires TGFβ inhibition, mediated by RA signaling, to conduct outgrowth and extension of the primary lung buds [40].

RA signaling machinery is present in the foregut at the beginning of lung organogenesis. At E9.5, *raldh2* is highly and ubiquitously expressed in the anterior foregut, specifically in the mesenchyme neighboring the prospective trachea and lung primordia, suggesting that RA is being synthesized. Moreover, at this gestational age, a high local RAR activation is also detected in all layers of the foregut where trachea and primordial lung are emerging. This expression pattern is consistent with the hypothesis that RA acts on its receptors present in the epithelium in early lung development. At E10, *raldh2* levels are preserved in the tracheal mesenchyme and proximal lung but decrease towards the distal lung. Conversely, *cyp26* is not detected between E9.5–10; consequently, at these stages, RA synthesis is not compensated by its degradation, pointing to a crucial role for RA in the formation of the lung bud primordium. It is worth noting that, as lung morphogenesis proceeds, *cyp26* is progressively expressed, therefore controlling RA levels (please refer to Section 3.2) [41].

Disruption of RA signaling in in vitro mouse foregut cultures impairs lung bud initiation [42]. Likewise, *raldh2^−/−^* knockout mice and dams exposed to severe vitamin A deficiency during gestation display lung agenesis [43,44]. In vitro studies revealed that RA induces *fgf10* expression in the foregut mesoderm, where lung initiates, which then activates FGFR2 signaling in the endoderm and induces primary bud morphogenesis [42]. By further dissecting *raldh2^−/−^* knockout mice, it was uncovered that RARβ mediates *fgf10* induction by RA. Differential activation of RARα and RARβ has opposite effects in *fgf10* mesodermal expression; nonetheless, both receptors are needed for proper lung development [44]. Furthermore, it has been shown that endogenous RA acts via WNT and TGFβ signaling to control *fgf10* expression. RA downregulates TGFβ activity in the foregut mesoderm, thus allowing local expression of *fgf10*, which is crucial for primary lung bud induction [40]. Conversely, RA facilitates the activation of WNT signaling, crucial for the appearance/maintenance of the respiratory field, by inhibiting the expression of a WNT/β-catenin antagonist (Dickkopf1) [45]. Hence, an RA-dependent fine-tuned equilibrium of the WNT/TGFβ/FGF10 axis is mandatory to permit both respiratory progenitors’ expansion and lung primordium formation.

RA acts in both endoderm and mesoderm to provide a niche for lung induction by regulating the expression of signals that will induce the lung and the competence to respond. In early somite stages, RA pre-patterns the lateral plate mesoderm and establishes the anterior foregut region that will form the respiratory niche. Subsequently, RA signaling promotes endodermal Hedgehog (HH) expression, which, mediated by mesenchymal GLI transcription factors, is indispensable for *wnt2/2b* and *bmp4* expression; subsequently, *wnt2/2b* and *bmp4* act on the endoderm to promote *nkx2.1* expression. The RA-HH-WNT signaling cascade that coordinates respiratory lineage specification is conserved between frog, mouse, and human [46]. Moreover, RA/RAR activity regulates the competence of the endoderm to activate the NKX2.1^+^ respiratory program in response to mesodermal WNT and BMP, independently of HH, during early somite stages of development.

Recently, it has been shown that RA signaling has different roles during endoderm organogenesis, acting in a distinct temporal and spatial pattern. In early gastrula stages (mouse E6–7.5), during endoderm formation, RA induces hindgut and pancreatic stages but inhibits foregut fate. Conversely, in early somitogenesis (mouse E7.5–8.5), during endoderm patterning, RA suppresses pharyngeal and promotes respiratory fate in the anterior endoderm. Finally, lung induction (mouse E9.5) relies on the RA/WNT/BMP axis previously described [46]. However, RA actions are only possible because, during the gastrula stage, WNT/BMP signaling specifies endodermal competence domains and, consequently, how cells respond to signals during the subsequent phases [47].

### 3.2. Pseudoglandular Phase

Lung branching morphogenesis occurs during the pseudoglandular phase (E12–16.5 mouse; E13–E18.5 rat; 5–17 weeks, human), and it is characterized by reiterative splitting of the airway epithelium into the surrounding mesenchyme [48]. The development of new generations of terminal buds contributes to defining the airway conducting system. This process is tightly regulated by a network of signaling cascades that operate via epithelial-mesenchymal interactions [30]. The RA pathway has been recognized as a critical regulator of pulmonary branching [4,41,49,50]. In fact, in *rdh10^−/−^* mutants, primary lung bud growth is arrested and branching morphogenesis impaired, thus preventing the formation of the lung [51]. Likewise, *raldh2^−/−^* mouse embryos display defective growth and branching, resulting in hypoplastic lungs [43].

RA signaling machinery is dynamically expressed during branching morphogenesis. For instance, *stra6* mouse transcript is present in the mesenchyme surrounding the bronchi [52]. Conversely, as lung branching initiates, *crbp* transcripts are present in both epithelial and mesenchymal compartments, and later its expression is restricted to the mesenchyme [53]. In the embryonic mouse lung, *raldh2* is expressed along the mesothelium surface at sites of low branching activity. By comparing *fgf10* and *raldh2* expression patterns, it is possible to observe that regions, where *fgf10* is expressed, are low in *raldh2* [41,54]. Conversely, *raldh1* mRNA is present in the proximal region of the bronchi [41,49], while *raldh3* is weakly expressed in the epithelium of the main bronchus [16]. Importantly, RA bioavailability must be balanced to avoid detrimental concentrations. As secondary buds start to emerge, *cyp26* expression displays a proximal-distal epithelial gradient with high expression at distal sites. In the mesenchymal compartment, *cyp26* is present between the secondary buds contributing to the regulation of RA levels in this region [41].

Retinoic acid signaling response is, ultimately, transduced through RAR and RXR nuclear receptors that recognize RARE sequences in the promoter region of RA-target genes. The importance of RAR is particularly evident in *rarαβ2* mouse mutants, which exhibit lung agenesis and hypoplasia due to the absence of left lung budding and altered branching morphogenesis [55]. In the developing lung, *rarα1* and *rarα2* are ubiquitously expressed [49,56], whereas *rarβ* expression is restricted to the proximal bronchi epithelium and the proximal subepithelial and subpleural mesenchyme [41,49,53]. In opposition, *rarγ* transcripts are faintly and ubiquitously expressed in the embryonic lung; indeed, RARγ knockouts do not affect lung branching [41,49,53,56,57,58]. Throughout branching, RARE-lacZ reporter activity is detected in the proximal bronchial region with a pattern that strongly overlaps *rarβ* expression. Moreover, RARE-lacZ lacks from both the distal bud epithelium and the mesenchymal compartment [41]. Furthermore, RXR can be sequestered by Chicken Ovalbumin upstream promoter-transcription factors (COUP-TFs), resulting in RA signaling inhibition. *coup-tfII* expression is found in the lung mesenchyme, particularly higher in the mesenchyme between the secondary buds, but absent from the pulmonary epithelium [41]. Recently, RA signaling was characterized in the embryonic chicken lung [50]. For instance, *stra6* is expressed in the periepithelial mesenchyme surrounding the epithelium as it occurs in the mouse lung [50,52]. *raldh2*, *raldh3*, *cyp26a1*, and *rarα* expression patterns are comparable to their mammalian counterparts. Curiously, *rarβ* has a stage-dependent expression that is present in the proximal region of the mesenchymal compartment [50]. It is worth mentioning that while in the mammalian lung RA signaling members are distributed between the mesenchymal and epithelial compartments, in the chick lung, all members seem to be restricted to the mesenchyme [50]. Nonetheless, transcripts like *stra6* and *raldh2* display highly conserved expression patterns. Moreover, the complementary expression pattern between *raldh2* and *fgf10* is conserved, with *raldh2* being expressed in the ventral region, and *fgf10* in the dorsal pulmonary region [50,59].

RA signaling must be finely tuned so that mammalian lung branching morphogenesis may occur. In the mesenchymal compartment, RA is produced in the mesothelial region, at low branching sites, by RALDH2. Conversely, RA synthesis is regulated by CYP26 and COUP-TFs in the mesenchyme between the secondary buds [41]. A gradient of RA is produced from the pleura to the periepithelial mesenchyme that surrounds the distal region of the growing bud. RA availability regulates *fgf10* that is typically expressed in the mesenchyme surrounding the distal bud tips [41,54]; for this reason, *raldh2^−/−^* mouse embryos phenotype is rescued by RA and FGF10 supplementation [43]. Concurrently, in the epithelial compartment, RA is produced in the proximal bronchi by RALDH1 [41,49] and may act through RARβ, which is detected in the same region [41,49,53]. RA epithelial levels are regulated by CYP26, which displays a gradient from the distal to the proximal region of the epithelial bronchi [41]. Intriguingly, *stra6* expression in the periepithelial mesenchyme, surrounding the epithelial bronchi, may play a role in retinol trafficking between epithelium and mesenchyme [52].

The role of RA signaling during pulmonary branching was uncovered by performing RA stimulation/inhibition studies. Mouse lung explants exposed to RA display an increase in lung branching in a dose-dependent manner [60]. Furthermore, RA supplementation can increase pulmonary branching in nitrofen-induced hypoplastic lungs, which display decreased RALDH2 activity [61]. Similarly, rat lung explants treated with exogenous RA exhibit an increase in the number of peripheral airway buds, epithelial perimeter, and the total area of the lung [62]. Likewise, RA supplementation, in the chicken model, revealed a dose-dependent increase in lung branching [50]. Furthermore, RA-exposed lung explants can display morphological alterations that resemble a more immature branching configuration; specifically, the formation of typical distal bud-like structures is impaired, promoting a more proximal-like phenotype [41,63,64]. Nevertheless, in some studies, a reduced number of terminal buds is observed in RA-treated lungs [41,56,63,64].

RA pathway modulation studies revealed a series of feedback control mechanisms underlying pulmonary branching morphogenesis. For instance, *crbpI* and *raldh1* expression are susceptible to be modulated by activation/inhibition of the RA cascade [49,56]. This feedback loop mechanism may regulate RA synthesis in the proximal epithelial buds [49]. On the other hand, RA supplementation increases both *rarβ* and RARβ but decreases RARα and RARγ [56,62].

RA downstream targets are not only signaling machinery components, but also members of other pathways. For instance, RA can directly downregulate *fgf10* expression or indirectly by stimulating the SHH pathway (through increasing *shh* expression) and, consequently, decrease *fgf10* transcript levels in the distal region of the lung [41,49,54,64,65]. Moreover, *bmp4* is also negatively modulated by RA signaling stimulation [41]. The interaction between RA, FGF, SHH, and BMP4 is crucial for branching and proximal-distal patterning [41,49,54,64,65,66,67]. In the proximal epithelium, RA modulates the expression of *foxa2*, a known regulator of epithelial differentiation and pulmonary branching [49,68]. RA pathway stimulation promotes *tgfβ3* expression in the lobar bronchial mesenchyme, pleura region, and bronchial epithelium [49], and downregulates *cftr* epithelial expression in the lobar bronchial and bronchial epithelium [49]. In fact, mammalian lung branching morphogenesis can be divided into two RA responsive centers: the distal epithelial region where *shh*, and *bmp4* are expressed and interact with *fgf10* mesenchymal expression; and the proximal epithelial region where RA regulates *tgfβ3*, *foxa2* and *cftr* [41,49,54,65].

There are other RA downstream targets described in the embryonic mammalian lung, namely *hoxa2*, *hoxa5*, *hoxb5*, and *hoxb6*, which are related to patterning. Moreover, RA regulates HOXB5 protein levels, and *hoxa2* and *hoxb6* proximal-distal patterns [64,69,70]. In addition, disruption of the RA pathway results in altered murine smooth muscle phenotype and a consequent increase in the expression of *acta2* and *myh11* [71]. During chick lung branching, RA supplementation does not affect *sox2* but decreases *sox9* expression (patterning-related genes); however, it affects *sox9* spatial distribution, which is progressively excluded from the distal regions of the lung. Still, the new distal branches do not exhibit proximal appearance [50,72,73]. Furthermore, RA signaling stimulation, of the embryonic chick lung, increases the expression of *rarβ*, *hoxb5*, and *meis2*; alters the spatial distribution of *cyp26a1*, *id2*, *tgfβ2*, and *sox9*; and has a very modest effect on *fgf10*, *fgfr2*, and *sox2*. Even though RA machinery is confined to the mesenchymal compartment, it can influence the expression of epithelial genes such as *shh*, *sox9*, and *fgfr2* [50]. In the chicken model, RA signaling stimulates early pulmonary branching and contributes to pulmonary branching proximal-distal patterning [50].

### 3.3. Canalicular Phase

The canalicular phase occurs between post-conception weeks 16 and 26 in human, E18.5–E20 in rat, and around E16.5–E17.5 in mouse [30]. During this phase, the epithelial airways continue to increase in size, and the terminal buds extend into the airspaces, forming the primitive pulmonary acini (terminal sacs) [74]. Simultaneously, distal mesenchyme, although still abundant at this phase, becomes progressively thinner. Additionally, early differentiation of AEC1 and AEC2 occurs, establishing an elementary gas exchange surface [25,75]; this respiratory region is morphologically different from the more proximal conducting airways [76,77,78]. Moreover, the bronchioalveolar duct junctions appear when epithelial differentiation happens and correspond to a stem cell niche; the location of this junction stays constant throughout life [79,80,81]. Finally, in this phase, vascularization begins. The continuous angiogenic process and juxtaposition of capillaries in close contact with the distal respiratory epithelium create the first air-blood barrier in the future alveolar ducts and saccules [82,83].

The role of RA in the canalicular phase has not been particularly dissected so far. However, in this phase, with the occurrence of cytodifferentiation of the columnar epithelium, the level of *rarα* transcripts decreases [84]. RA is a key regulator of cellular growth, proliferation, differentiation, and embryogenesis per se, and one cannot discard that it may contribute to this phase of lung morphogenesis. For instance, retinoic acid is a major regulator of angiogenesis and vasculogenesis [85], and the organization of the pulmonary vascular bed initiates during the canalicular period. Therefore, RA may be involved in the development of the lung vascular system (please refer to Section 3.6).

### 3.4. Saccular Phase

The pulmonary saccular phase is characterized by the widening and growing of the terminal airways, which contribute to forming clusters of larger airspaces called saccules. During this phase (weeks 24–38 in human; E20–P4 in rat; E17.5–P4 in mouse), the enlargement of the future gas-exchange region causes condensation of the mesenchyme in-between airspaces, establishing the primary septa. Primary septa are composed of a double layer of capillaries separated by a central layer of mesenchyme. The majority of the primary septa surface is covered by AEC1, and the remaining surface is occupied by AEC2. Throughout this phase, lamellar bodies appear, and surfactant secretion initiates. Smooth muscle cell precursors start to form a network of elastic fiber and collagen fibrils that prepare the lung for alveolarization [30,86,87].

*rar* transcripts are present in the embryonic rat and mouse lung during the canalicular phase [84,88]. The expression levels of *rarα* transcript decrease in this phase [84], which seems to be required to allow sacculation and the differentiation program that contributes to forming mature AEC1 [88]. On the other hand, *rarβ* expression increases significantly in the late saccular period. This increase in *rarβ* matches AEC1 and AEC2 induction, suggesting a role for *rarβ* in preparing the lung for the alveolar phase. Low expression levels of *rarγ* were detected in this period [84]. Such findings point to a crucial role of the RA signaling pathway in preparing the lung for sacculation, and later, during the saccular phase, to prepare the lung for alveolarization.

### 3.5. Alveolar Phase

Alveologenesis or alveolarization is the process by which the gas exchange functional units, the alveoli, are generated. Alveoli formation is mainly a postnatal event, and it is time and species-dependent. For instance, mammals like deer, guinea pigs, or sheep are born with functional alveoli; mother-dependent mammals like mice, rats, and humans are born with primitive alveoli and, so, alveologenesis continues postnatally [89]. In mouse and rat, alveologenesis occurs entirely in the postnatal period, approximately from P4 to P36 and P4 to P60, respectively [23,25,30]. In humans, part of the alveoli is formed before birth, but their genesis continues postnatally. Alveologenesis is divided into three phases: classical alveolarization (week 36 until ∼3 years), microvascular maturation (week 36 until young adulthood), and continued alveolarization (∼2 years until young adulthood) [30].

Throughout the canalicular and saccular phases, the terminal tubes are transformed into small saccules separated by the primary septum. During classical alveologenesis, the sacs are subdivided by the ingrowth of secondary septum, the outline of the future alveoli. Myofibroblast progenitors, endothelial cells, fibroblasts, and lipofibroblasts (LIFs) cover the secondary septa, and matrix proteins such elastin are deposited at the tip [23,25,90,91]. At first, alveolarization is quite fast (known as “bulk alveolarization”), but then, this process is slowed down, giving rise to the continued alveolarization. As the alveoli mature, the surrounding capillaries are remodeled, forming the capillary unit, and the endothelial cells are located in the proximity of the AEC1 cells permitting an efficient gas exchange. This process is known as microvascular maturation and occurs simultaneously with alveolarization [23,25,30,90,91,92,93].

Secondary septation is intimately associated with several signaling events. For example, platelet-derived growth factor α (PDGFα) regulates myofibroblast differentiation and elastin production; TGFβ regulates elastin expression [90,91,94], whereas ephrin B2 plays an essential role in endothelial cells. Furthermore, *fgfr3*/*fgfr4* double null mice fail to generate the secondary septa pointing to a role for FGF signaling in controlling this event [95]. Finally, RA is crucial for proper alveolar formation [23,25].

Alveologenesis in the developing lung is partially regulated by vitamin A [96], and endogenous RA is present in the postnatal lung [97,98]. Retinoids have been recognized over the years as alveolar morphogens and possible therapeutic mediators [99]. Different studies have demonstrated that vitamin A deficiency induces major histological changes in the lung, namely: airspace enlargement, thinner alveolar walls, more alveolar breaks, and an increase in the mean free distance in the air spaces [100]. Furthermore, vitamin A deficiency impairs lung epithelial function in rodents and promotes bronchopulmonary dysplasia (BPD) in humans [101,102]. The precise mechanism by which RA regulates alveolar formation remains poorly understood, although RA regulation of myofibroblast proliferation was shown to rely on intact FGF signaling [25]. Conversely, RA supplementation in explant culture induces septation, and retinol treatment of premature lambs promotes alveologenesis [103].

RA signaling machinery is differentially expressed during alveologenesis. For instance, in mice, CRBPI is present in the alveolar walls and exhibits higher expression levels around P9, whereas CRBPII is detected during alveolar formation at P4 [97]. Furthermore, in the rat model, CRABP levels rise shortly after birth, reach its maximum values at P10, and at P21 it is barely detected. These genes were upregulated during alveologenesis in whole rat lung, and isolated LIFs [104], and the alterations in the expression levels coincide with the morphological transformation of primitive saccules into differentiated alveoli [105,106]. Likewise, RALDH1 and RALDH2 are expressed in a temporal and spatial regulated manner in the lung and are associated with different patterns of alveolar cell wall proliferation. For example, in mice, RALDH1 levels are higher at P4, and it is found in LIFs populating alveolar walls, like CRBPI, and in the bronchial epithelium. Conversely, RALDH2 peaks just before birth, it slowly declines during alveologenesis, and it is expressed in the bronchial epithelium and pleural mesothelial cells but not in the alveolar walls [107]. Secondary septation is RALDH/RA-dependent. Epithelium-derived vascular endothelial growth factor A (VEGFA) regulates RALDH levels and, consequently, RA production by endothelial cells. RA acts as a paracrine factor to induce the expression of FGF18 by lung fibroblasts that, on its turn, regulates elastin deposition [108].

RA and RAR receptors are crucial for septation and secondary crest formation [94]. RARα, RARβ, and RARγ were identified in rat lung isolated LIFs and in postnatal mice lung, namely RARα1, RARβ2, RARβ4, and RARγ2 [107]. RARs are present in the alveolar walls, bronchial epithelium, pleura, and bronchial vascular smooth muscle. Their expression changes with time and, in mouse, increases significantly at P4 [97]. Lungs from *rarα^−/−^* mice are similar to wild type at P14 (end of septation), but, at P50, the alveoli number and surface area are decreased. These results hint that RARα is not vital for alveolar septation, but it may have a role in later phases of alveologenesis [109]. Conversely, increased RA signaling of a dominant active RARα receptor in the epithelium leads to lung immaturity [88]. In *rarβ^−/−^* mice (exon 10 deletion), septation occurs earlier and faster; also, the administration of RARβ specific agonists in the rat model impairs septation. Overall, it seems that RARβ negatively controls the septation process [110]. However, RARβ seems to be important for neonatal alveolar growth, as disclosed by studying a different *rarβ^−/−^* mutant (exon 6 deletion) that displayed a decreased surface area of gas exchange [111]. RARγ is required for alveoli formation during the first 28 postnatal days. *rarγ^−/−^* display a decrease in the elastic tissue of the whole lung, and the alveoli number. In contrast, the distance between the alveolar walls and the mean chord length increased. Additionally, in lipofibroblasts, the trophoelastin mRNA decreased at P12 [94]. Moreover, RARγ agonist’s administration promotes alveolar repair in an animal model of emphysema [112]. Altogether, it seems that RARγ positively regulates pulmonary septation. Furthermore, in cultured LIFs, it was demonstrated that RARβ and RARγ increase at birth, which corresponds to the peak of RA storage. The increase in RARγ is temporary since it decreases after P2 (RA in the LIFs also decreases after postnatal day 2). In summary, RA may act at RARγ level in the early postnatal period [107]. RAR heterodimerizes with RXR before binding to DNA. Homozygous null RXR-α mice die in utero. Additionally, null for RARγ and heterozygote for RXR-α present decreased elastin expression in the LIFs at P10; and a decrease in pulmonary elastic tissue in the alveolar septa and not at the airway or vascular walls at P28 [94].

After the administration of retinyl palmitate to pregnant rats, the levels of retinyl esters increase in the fetal and postnatal lung [113], which implies that fetal lungs store retinol or retinyl esters from the progenitor. The lung is the second biggest storing place of retinoids following the liver [114]. LIFs are the main retinoid reservoir of the lung and contain all the components of the pathway [104,114,115]; consequently, they can uptake circulating retinol or hydrolyze stored retinyl esters and convert them into retinol or RA [116,117]. LIFs are localized in the septating tissue adjacent to AEC2 [118]. LIFs generate all-trans retinoic acid (ATRA) that acts autocrinally to upregulate elastin gene expression and synthesis, thus playing a crucial role in the induction of septal eruption [116,119,120]. Moreover, ATRA acts paracrinally to induce proliferation of the adjacent AEC2 [121,122], gene expression [115], and angiogenesis in microvascular endothelial cells [123]. After birth, esters start to disappear from the lung and are converted into active forms, like retinol and RA [98,104,124]. Retinyl esters are abundant in the last three prenatal days (similar amount comparing to the liver) [117], and its depletion correlates with lung maturation [98]. Retinoid clearance is associated with changes in the epithelium of the conducting airways. Specifically, cell phenotype alters from stratified columnar epithelium with goblet cells to keratinizing squamous epithelium [125].

RA regulates positively or negatively the expression of many genes. Among them, RA is important for the synthesis of the surfactant proteins SPA, SPB, and SPC. Administration of retinyl palmitate at high doses to pregnant rats elevates both total phospholipid and the desaturated portion of phosphatidylcholine of the pulmonary surfactant. However, SPA concentration remains unchanged. Additionally, repetitive RA administration increases surfactant phospholipid content [126]. On the other hand, RA also has inhibitory actions. In some studies, it was shown that RA decreases the expression of both mRNA and protein levels of SPA [127].

Regenerative processes are intimately connected with the reawakening of developmental programs [128], and RA has been described as important in alveolar regeneration. Exogenous ATRA, RARα and RARγ agonists and 4-oxo RA can induce alveolar regeneration in adult rats displaying emphysema and partially rescue their phenotype [110,112,129]. On the contrary, pan RXR agonist, 13-cis RA, and retinol were not regeneration inducers, and RARβ was not required for regeneration [130]. In some studies, ATRA was shown to induce apoptosis, a normal process in wound healing, in rats previously treated with elastase, rescuing the alveolar surface area whereas, in others, RA did not affect lung function [131,132]. Recently, using lung organoid models, it was shown that RA pathway stimulation led to a decrease in the organoid size and inhibited epithelial proliferation. In contrast, RA pathway inhibition promoted epithelial proliferation in mice lung organoids and human organoids from chronic obstructive pulmonary disease (COPD) patients. In the mouse model, increased proliferation happened with concomitant suppression of epithelial differentiation from the airway and alveolar epithelium. Furthermore, cell proliferation was intermediated by YAP (yes-associated protein) activation and FGF signaling. Lastly, the inhibition of Histone deacetylase in combination with ATRA was proposed as a potential method to restore adult lung epithelial cell differentiation [133]. An in silico study provided the first clues regarding the possible role of ATRA in minimizing inflammation in elastase-induced emphysema in rat lungs and, therefore, in alveolar epithelium regeneration. It was proposed that ATRA can bind to receptors and ligands of both ERK and JAK-STAT signaling pathways, namely, TNF-α, IL6ST, TNFR1, and IL6. Furthermore, ATRA showed more binding efficiency for TNF-α and IL6ST and can potentially regulate both ERK and JAK-STAT pathways by acting at its first steps. ATRA administration restored lung histology, the proteases/antiproteases balance (imbalance is characteristic of emphysema condition), the levels of inflammatory markers and antioxidants, and the expression of candidate genes of ERK and JAK-STAT. In conclusion, ATRA reduces inflammation and improves alveolar epithelium regeneration in rat lung with emphysema [134].

### 3.6. Vascular Development

The pulmonary vasculature forms synchronously with the airways by a process called distal angiogenesis [135]. Actually, disruption of lung vascular development impairs airway and alveolar formation, a feature that characterizes some congenital lung diseases [136]. VEGFA plays a crucial role in the interaction between the epithelial and endothelial compartment [137]. On its turn, new endothelial cells are bordered by pericytes that require PDGFβ signaling [138]. As it occurs with airway formation, fine-tuning of multiple signaling pathways is essential for vascular development [136].

RA signaling regulates the proliferation of endothelial cells during vasculogenesis in mice embryos and yolk sacs [139]. In the particular case of the lung, Schmidt et al. [140] suggested that prenatal administration of RA improves lung vascularization and VEGF expression in a rat model of congenital diaphragmatic hernia (CDH). Later in development, during alveologenesis, endothelial cells produce RA that promotes FGF18 expression in adjacent mesenchymal cells, thus regulating elastin production. Simultaneously, RA acts autocrinally controlling endothelial cell proliferation and tube formation [108]. More recently, it has been demonstrated that RA signaling is involved in pericyte migration, angiogenesis, and collagen IV expression [141].

## 4. RA and Lung Disease

Retinoic acid is a key regulator of pulmonary organogenesis and homeostasis by orchestrating the different phases of lung development. Impairment of RA signaling has catastrophic effects on the development of the respiratory system. Such defects include lung hypoplasia, pulmonary agenesis, lack of esophageal-tracheal septum, and congenital diaphragmatic hernia (CDH) both in animal models and in humans [4,55,142,143]. Dietary vitamin A deficiency (VAD) during pregnancy represents a major concern in developing countries who do not have access to well-balanced food intake and, consequently, are more prone to develop RA-associated fetal lung diseases [3]. The genetic burden, particularly the presence of mutations in the RA machinery, also contributes to developing more or less dramatic lung phenotypes.

In the particular case of CDH, which is characterized by a spectrum of lung hypoplasia and consequent pulmonary hypertension, several studies reported a defective RA signaling [61,143]. CDH has been observed in infants with decreased levels of serum retinol and RBP; and in individuals with deletions in chromosome 15q, where the RA signaling machinery components are located. Moreover, *rarαβ2* knockout animals display unilateral lung agenesis, contralateral lung hypoplasia, and diaphragmatic defects, which are features of CDH [55,142,143,144]. Furthermore, vitamin A/RA supplementation has a very positive effect on nitrofen-induced rat model of CDH (inhibition of RALDH2). In this case, RA pathway stimulation reduces CDH incidence, improves lung hypoplasia, vascular abnormalities, and, consequently, lung development [143].

Chronic respiratory disorders such as pulmonary fibrosis, lung cancer, emphysema, and COPD, are also VAD-related. Likewise, under VAD conditions, childhood asthma and respiratory infections are promoted [3,145]. On the other hand, retinoids can be applied to promote alveolarization of premature infants at risk of bronchopulmonary dysplasia; or to stimulate the alveolar development and de novo surfactant production of CDH-associated pulmonary hypoplasia [146,147,148].

## 5. Final Remarks

The importance of RA signaling during embryonic development and, specifically, during pulmonary organogenesis is indisputable. From lung specification to alveologenesis, RA signaling fine-tunes the epithelial-mesenchymal interactions that trigger the formation of a fully functional organ. With this review, we aimed to describe the current knowledge regarding the role of RA in the distinct phases of lung development. Alterations in this signaling pathway cause major developmental defects, but that, by itself, would be a topic for an extensive review. Table 1 summarizes: (1) the specific RA components/crosstalk involved in each of the phases; (2) the phenotype when RA components are silenced, demonstrating its involvement in the etiology of some developmental lung diseases. Moreover, and considering retinoic acid role in the later phases, RA might be a suitable candidate to improve some chronic diseases in the adult by promoting regenerating/repair events.

## Figures and Tables

**Table 1:** Summary of retinoic acid components, crosstalk, and knockouts during lung development.

Lung Developmental Phase	RA Pathway Machinery	RA Pathway Targets	Knockouts with Lung Phenotype	References
Embryonic	*cyp26* *raldh2* RARα, β, γ	*fgf10*, FGFR2 WNT, *wnt2/2b* TGFβ HH *bmp4* *nkx2.1*	*raldh2^−/−^* (lung agenesis)	[40,41,42,43,44,45,46,47]
Pseudoglandular	*stra6* *crbp* *raldh1*, *2*, *3* *cyp26* *rarα1*, *α2*, *β*, *γ* *coup-tfII*	*fgf10*, *fgfr2* *shh* *bmp4* *foxa2* *tgfβ2*, *β3* *cftr* *hoxa2, a5, b5, b6* HOXB5 *acta* *myh11* *sox2, sox9, id2* *meis2* *crbpI* *raldh1* *rarβ,* RARα, *β*, γ	*rdh10^−/−^* (absence of lung, primary lung bud growth arrested and branching impaired) *raldh2^−/−^* (hypoplastic lungs, defective growth and branching) *rarαβ2^−/−^* (lung agenesis and hypoplasia, absence of lung budding, altered branching)	[16,41,43,49,50,51,52,53,54,55,56,57,58,62,64,69,70,71]
Canalicular	RARα	-	-	[84]
Saccular	*rarα*, *β*, *γ*	-	-	[84,88]
Alveolar	CRBPI, II CRABP RALDH1, 2 RARα, β, γ RXR	VEGFA FGF signaling SPA, B, C, *spa* YAP TNF-α, TNFR1 IL6, IL6ST	*rarα^−/−^* (↓ alveolar number and surface area) *rarβ^−/−^* (early & faster septation; ↓ surface area) *rarγ^−/−^* (↓ elastic tissue, ↓ alveoli number, ↑ distance between the alveolar walls) *rxrα^−/−^* (embryonic lethality) *rarγ^−/−^*, *rxrα^+/−^* (↓ elastic tissue at the alveolar septa)	[88,94,97,104,107,108,109,110,111,112,123,126,127,130,134]

↓, decrease; ↑, increase.
